# Audit of stored strain energy and extent of future earthquake rupture in central Himalaya

**DOI:** 10.1038/s41598-018-35025-y

**Published:** 2018-11-12

**Authors:** K. M. Sreejith, P. S. Sunil, Ritesh Agrawal, Ajish P. Saji, A. S. Rajawat, D. S. Ramesh

**Affiliations:** 1 Space Applications Centre (ISRO), Ahmedabad, India; 2 Indian Institute of Geomagnetism (DST), Mumbai, India; 3 Department of Marine Geology and Geophysics, Cochin University of Science and Technology (CUSAT), Kochi, India

## Abstract

The deadly 25 April 2015 Gorkha earthquake (M_w_ = 7.8) and aftershocks have partially released the accumulated interseismic strain along the Main Himalayan Thrust (MHT). Postseismic deformation associated with this earthquake is mainly confined to the north of the rupture. This suggests possible occurrence of future large events towards west or south, where MHT is locked. Asperities arising due to heterogeneity in the stress-strain patterns are believed to play a major role in controlling the coseismic rupture propagation. We determine interseismic coupling along the MHT and spatial variations in total strain rate using two decades of GPS, InSAR and sprit leveling data. Further, b-values derived from the seismicity data are used to identify zones of stress accumulation. We demonstrate that the 2015 earthquake ruptured an asperity which hosted high strain and stress accumulation prior to the event. A similar asperity towards west of the epicenter with unreleased strain energy is identified. This could spawn a future large earthquake akin in magnitude to the 2015 Gorkha event. These findings compel a revisit of the seismic hazard assessment of the central Himalaya.

## Introduction

The 25 April 2015, Gorkha, Nepal earthquake of magnitude M_w_ 7.8 (hereafter referred as 2015 Gorkha earthquake) ruptured the Main Himalayan Thrust (MHT), a detachment boundary between the Indian and Eurasian Plates, which absorbs roughly half the India-Eurasia convergence (~40 mm/yr). The shallow portion of the MHT, beneath the lesser Himalaya is locked for about 100 km from the Main Frontal Thrust (MFT) accumulating elastic strain energy, while the deeper portion is creeping beneath the higher Himalayas. The elastic strain energy stored during the interseismic period is released periodically due to earthquakes and causes ruptures along the locked upper part of the MHT system^1^.

**Figure 1:**
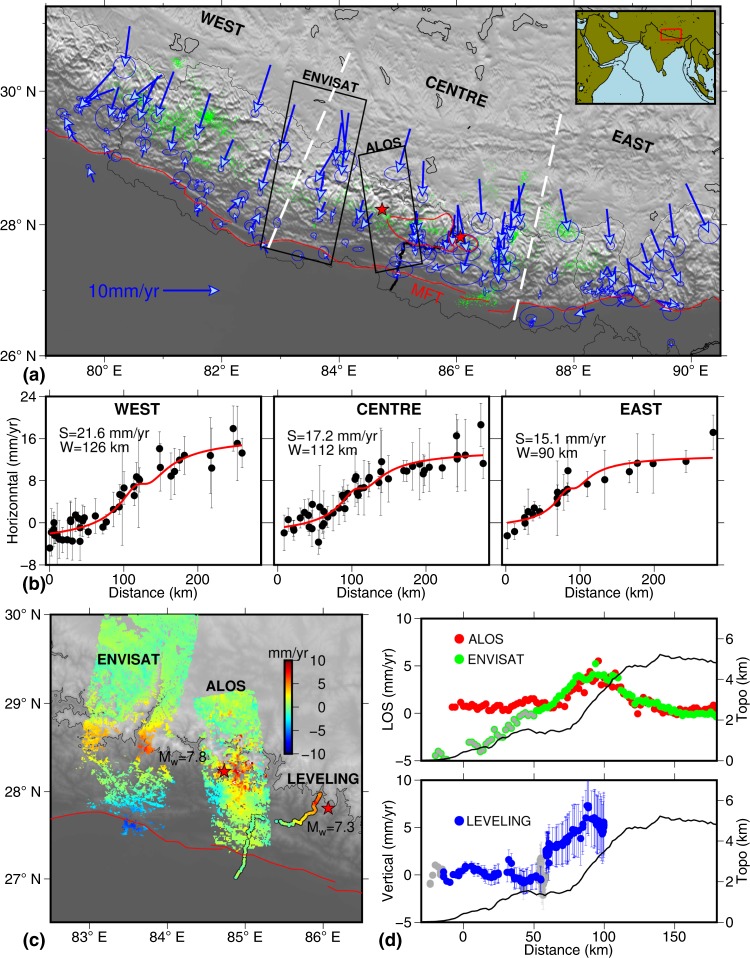
Convergence and vertical deformation along the central Himalaya from geodetic observations. (**a**) Topography of the Central Himalaya with horizontal velocity from GPS data in Indian reference frame (blue) tipped with 68% confidence ellipse. White dashed line represents the boundary between West, Central and East segments. The epicenters of 2015 Gorkha and Mw = 7.3 aftershock (red stars) and their coseismic rupture areas with slip > 2m (red lines) are shown. Microseismicty data is shown as green dots. The Tracks of ENVISAT^14^, ALOS and spirit leveling^15^ is shown in black. Location of Main Frontal Thrust (MFT) is shown as red line. (**b**) Horizontal GPS velocity projected along the arc perpendicular profiles (black dots with error bar) along with best-fit forward models (red lines). (**c**) Maps of InSAR and spirit level data. (**d**) LOS velocity derived from InSAR and vertical velocity from spirit leveling data projected along arc perpendicular swaths. InSAR and leveling data marked with gray dots are excluded from the analysis.

The coseismic rupture produced by the 2015 Gorkha earthquake travelled east of the epicenter and failed to breach the surface towards south to the MFT. The largest aftershock which occurred on 12^th^ May 2015, about 150 km east of the main shock, ruptured further the easternmost part of the main event rupture area (Fig. 1). Coseismic and postseismic deformations due to the 2015 Gorkha event are well documented by several researchers using Synthetic Aperture Radar Interferometry (InSAR) and Global Positioning (GPS) data^2–9^. Joint inversion of these geodetic data have provided detailed slip distribution pattern on the causative fault during the coseismic and postseismic periods. It is interesting to note that the postseismic slip is confined totally to the north of the rupture and occurs over the deeper portion of the MHT. This scenario suggests increased risk of future earthquake occurrence on the locked portion of lesser Himalaya both up-dip and towards west.

The rupture propagation of plate boundary earthquakes, particularly the great Himalayan earthquakes are believed to be controlled by asperities and associated structural complexities^10,11^. However, geodetic mapping of asperities along the Himalayan arc remains a challenge owing to poor model resolution at larger depths imposed by sparse data coverage^12,13^. In this study, we estimate the interseismic deformation and convergence pattern of central Himalaya using a compilation of geodetic data including new InSAR measurements along a track passing through the epicentral region of the 2015 Gorkha earthquake. Variations in the surface strain prior to this earthquake occurrence are mapped utilizing GPS data. While, the corresponding stress fields are based on b-values derived from micro-seismic data recorded during 1994–2009 by the local network of National Seismological Centre (NSC) in Kathmandu, Nepal. Our study reveals existence of two clear asperities delineated by high strain and stress towards west and east of the 2015 Gorkha epicenter. The eastern asperity ruptured during the Gorkha earthquake leaving the western part as locus of unreleased strain energy to spawn an impending earthquake.

## Himalayan Convergence and Interseismic Coupling along MHT

GPS derived velocities from 36 continuous stations of Nepal Geodetic network along with published data from the neighbouring regions provide a dense coverage of the area between eastern part of the Kumaon Himalaya and western Bhutan (Fig. 1, Supplementary Fig. S1, Supplementary Table S1). The GPS derived horizontal velocities suggest about ~15–21 mm/yr convergence across the central Himalayan arc. We present a new InSAR derived deformation map obtained from ALOS- PALSAR-1 data along a track through the epicentral region of the 2015 Gorkha earthquake (Fig. 1, Supplementary Fig. S2). The InSAR derived deformation pattern primarily represents the vertical component, as sensitivity to horizontal deformation is extremely poor due to the satellite acquisition geometry. The vertical deformation across the Nepal Himalaya is about 5–8 mm/yr. This compares well with those obtained from the ENVISAT InSAR data towards west^14^ and spirit levelling measurements^15^ towards east of the ALOS track (Fig. 1). The ENVISAT interferogram has large-scale unwrapping errors close to MFT and depicts ambiguous subsidence values. Whereas the new ALOS-1 data, which are more reliable, show excellent agreement with the spirit levelling measurements. Peak uplift rates derived from the InSAR and level data coincide with the 3500 m topographic contour and match with the region of maximum gradient of horizontal deformation derived from GPS measurements (Fig. 1c).

Based on the surface velocity and geometry of the MFT, the horizontal velocity field is projected along three orthogonal profiles normal to the Himalayan arc representing the western (78.4°E to 82.4°E), central (78.4°E to 82.4°E) and eastern (86.6°E to 88.1°E) segments. We use a simple elastic dislocation model^16^ to estimate slip rate, dip and width of the locked zone. A grid search minimizing the *χ*^*2*^ of the misfit between the observed and model was used to estimate the model parameters (Fig. 1b, Supplementary Fig. S3, Supplementary Table S3). The western segment shows a relatively large slip rate (21.6 ± 1.7 mm/yr) compared to the central (17.2 ± 2.1 mm/yr) and eastern segments (15.1 ± 1.4 mm/yr). The width of the locked zone and dip are estimated as 90–120 km and 10°–12° respectively. The 2D model of GPS profiles clearly suggest presence of broad, first-order along strike variations in the slip rate along the MHT and are consistent with the recently reported results^17^.

**Figure 2:**
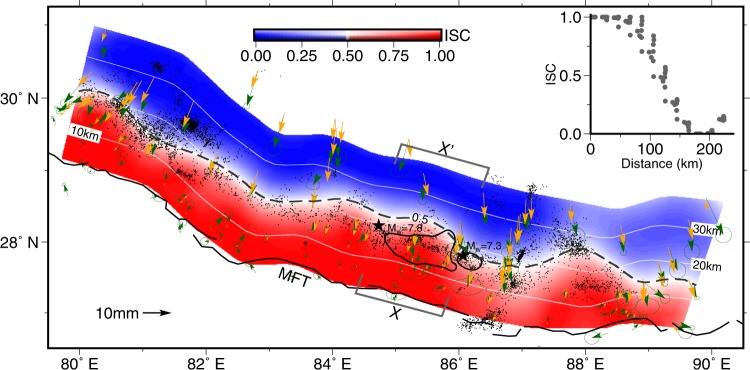
Interseismic coupling model of the central Himalaya. The observed and model GPS velocities are shown as green and orange arrows. The epicenters of 2015 Gorkha and Mw = 7.3 aftershock (black stars) and their coseismic rupture areas with slip > 2m (black lines) are shown. Microseismicty data is shown as black dots. ISC = 0.5 contour is shown as dashed line. ISC values along swath XX’ is shown as insert graph.

In order to estimate interseismic coupling (ISC), we invert geodetic datasets assuming linear superposition of rectangular dislocations in an elastic half-space^18^. The fault model assumes 10° dip and down-dip length of 270 km from MFT. The geometry of the MFT is approximated with 15 fault segments along the strike direction. ISC is computed as the normalized difference between the long term slip rate and the modelled fault slip rate (for details of inversion refer the section methods and Supplementary Figs. S4–7). From MFT about 100 km towards north, the ISC value ~1 suggests interseismic locking of the fault segment (Fig. 2) along the lesser Himalaya. However, a sharp down dip gradient in ISC from 0.75 to 0.25 within a distance of ~40 km can be noticed (Fig. 2). This coincides with the belt of mid-crustal seismicity and hence indicative of stress accumulation at the base of MHT. The derived ISC model did not reveal any obvious along-strike asperity. This is despite the ability of the model to resolve features on the order 70–100 km as suggested by the resolution tests (Supplementary Fig. S6). It is noteworthy that the 2015 rupture area and surrounding region (82.5°E to 86°E) effectively recover the input slip model, where InSAR and levelling data are available compared to the portions where only GPS data exist (Supplementary Fig. S6). Therefore, absence of along-strike asperities in the study region cannot be related to geodetic data distribution.

## Total Strain Rate

**Figure 3:**
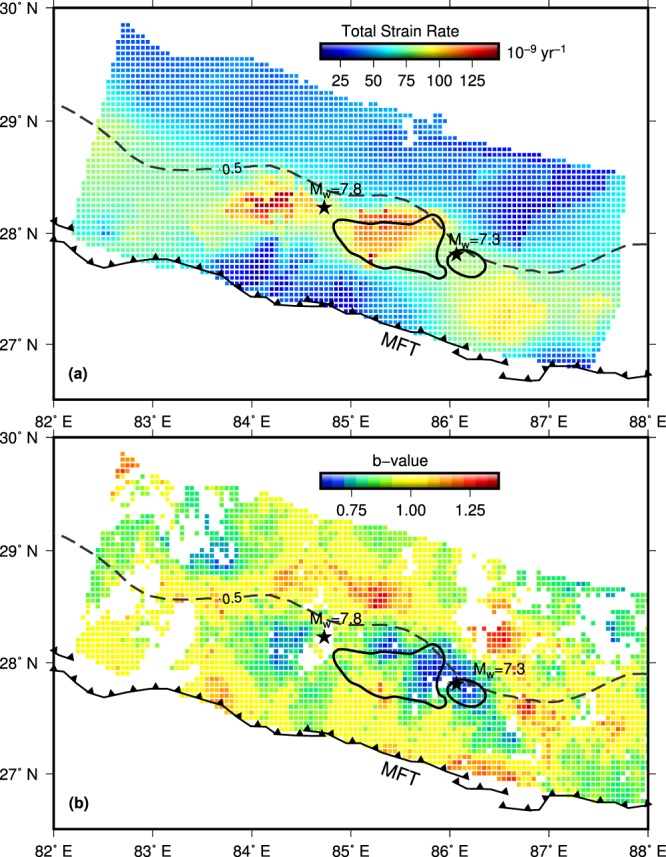
Comparison of total strain rate and b-value maps with rupture area of 2015 Gorkha earthquake. (**a**) Total strain rate derived using GPS data. (**b**) b-value map derived from microseismic data. (**a**,**b**) The epicenters of Gorkha and Mw = 7.3 aftershock (black stars) and their coseismic rupture areas with slip > 2m (black lines) are shown. ISC = 0.5 contour is shown as dashed line.

GPS derived horizontal velocities are used to generate the strain rate map of central Himalaya. In order to relate the deformation rate to seismicity and stress build-up during the interseismic period, we estimated the 2-D second invariant to the strain rate tensor independently from the principal strain components (Fig. 3a). The second invariant strain rate is equivalent to the Total Strain Rate (TSR) and could be related to spatial distribution of earthquakes^19,20^. TSR in central Himalaya appears to be highly segmented across the strike depicting high strain from MFT to the Higher Himalayas and low strain towards Tibet (Fig. 3a). The along strike high TSR mimics the belt of micro seismicity (Supplementary Fig. S8) and matches well with the locked portion of MHT (Fig. 3a). A low TSR seen for a portion closer to MFT, between 83.5° and 85.5°, appears to be an artifact plausibly caused by sparse GPS data distribution. This is also indicated by low Signal to Noise Ratio (Supplementary Fig. S8). Importantly, the total strain rate clearly depicts two patches of high strain accumulation zones, one towards east and the other west of the Gorkha earthquake epicentre (Fig. 3a). The eastern patch coincides well with the rupture area of the M_w_7.8 and M_w_7.3 events. However, the M_w_7.8 epicentre itself is located in a region of comparatively low strain rate.

## b-value and Differential Stress

The magnitude and frequency distribution of the earthquakes obey a power law, known as the Gutenberg-Richter law, and is expressed as *logN* = *a* − *bM*, where *N* is the number of earthquakes greater or equal to magnitude *M*; *a* and *b* are the constants indicating productivity level and size distribution respectively^21^. Global average of b-value is ~1, but in general this value shows significant variations between individual fault zones^22,23^ or even within a fault^24,25^. Laboratory experiments, numerical modeling and seismicity data conclusively suggest that b-values are negatively correlated with differential stress^26^. Large earthquakes are known to rupture asperities on faults having large tectonic stress as indicated by the pre-earthquake b-value maps^23,27,28^. We use seismicity data^29^ from the NSC recorded during 1994–2009 to map spatial variations in the b-value along the MHT (Fig. 3b, Supplementary Figs. S9 and 10). It can be observed that the deeper portions of MHT near the brittle-ductile transition zone^12^ are associated with significantly low b-values (b < 1) compared to the southern and northern portions. However, the most striking feature is the spatial variability along the MHT (Fig. 3b). The b-value map clearly reveals two patches of low regions (b < 0.75), one towards east and other to the west of the 2015 epicenter. The eastern asperity correlates well with the rupture area of the 2015 Gorkha earthquake. Schorlemmer and Wiemer^23^ reported that during the 2004 Mw 6.0 Parkfield, California earthquake, the areas of low b-values controlled most of the coseismic rupture and suggested that the low b-value region could be due to increase in strain energy before the earthquake occurrence. The excellent correlation obtained between the low b-value and high strain rate on MHT therefore clearly suggests localised increase in the strain energy.

## Locked Asperities, Partial Ruptures and Earthquake Hazard

**Figure 4:**
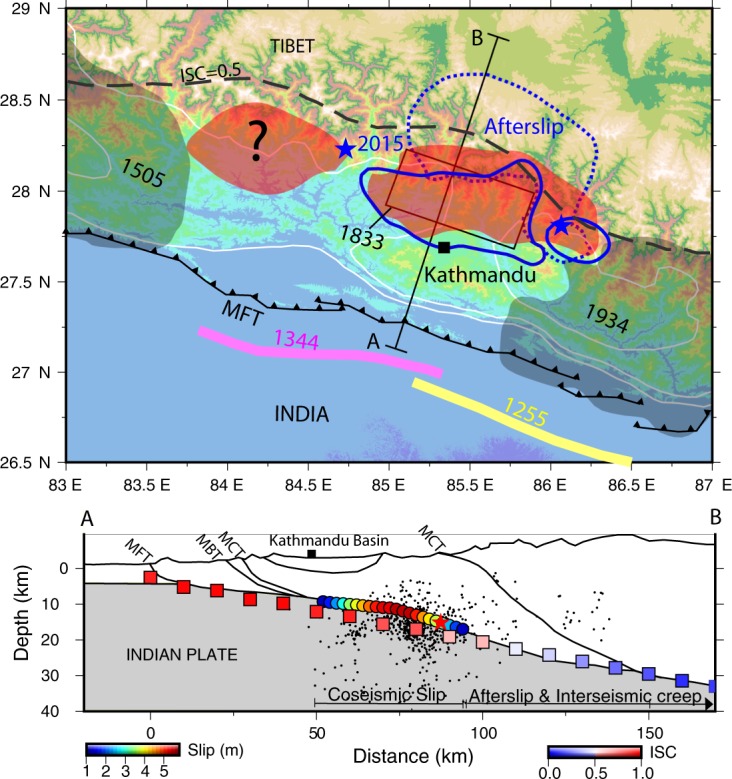
Asperities mapped in the present study and rupture zone of 25 April Nepal earthquake and historical events. Rupture zones of the 2015 earthquakes and afterslip regions^5,8^ are marked with blue continuous and dashed lines. Position of asperity mapped is shaded with red. Approximate rupture areas of the historical earthquakes of 1344, 1255, 1505, 1833 and 1934 are marked. Geological cross section along line AB with modelled of coseismic slip along the MHT^5^ is shown below. Microseismic data and mean ISC values along a swath ±75 km from the location of AB is also shown. The red star represent the projected location of 2015 Gorkha earthquake. MBT- Main Boundary Thrust, MCT- Main Central Thrust.

The elastic strain energy stored in the Himalaya is released through earthquakes with large ruptures reaching the surface or by incomplete ruptures. The cause for partial rupture could be the heterogeneity in the elastic energy distribution manifested as regions of strongly and weakly coupled plate interfaces^30^. However, the derived ISC pattern is fairly uniform along the central Himalayan arc and does not reveal presence of such heterogeneities (Fig. 2). On the contrary, the total strain rate and b-values together suggest presence of two asperities towards the west and east of the 2015 Gorkha earthquake epicentre (Fig. 3a,b). Considering b = 0.75 and TSR = 90 × 10^−9^ yr^−1^ as reference values, the approximate extent of these asperities are demarcated (Fig. 4, Supplementary Fig. S11). The eastern asperity culminated into the coseismic rupture of the 2015 event with no surface manifestation, albeit, the western counterpart is yet to be visited by an earthquake. This partial rupture induced by the eastern asperity warrants an explanation. One argument forwarded to explain the partial rupture, particularly the observed interruption of coseismic rupture of the 2015 earthquake towards the surface, is traced to the structural complexity of the MHT^5,31–33^. As evidenced from the mapped asperity, our results clearly suggest that the local heterogeneity in the stress/strain regime, instead, must have played a key role in controlling the coseismic rupture (Figs. 3a,b and 4). Numerical modelling of earthquake cycle, using rate and state frictional laws, suggests that the heterogeneity in fault frictional properties and preseismic shear stress are primarily responsible for the partial ruptures^34^.

We notice that the maximum slip region (>4 m) of the 2015 Gorkha earthquake correlates fairly well with high strain and stress (ie. low b-value) patches (Fig. 3a,b). While, its epicentre is associated with relatively low stress and strain rates. Therefore, together they corroborate heterogeneity in stress/strain energy distribution as the main controlling factors in rupture dynamics. This is consistent with the multi-stage rupture evolution proposed for the 2015 Gorkha earthquake^35^. The initial slow slip and delayed fast slip stages correlate with the weaker and stronger part of the mapped asperity respectively. Similarly, P-wave back projection results suggest that the beginning of the earthquake experienced a dynamic weakening mechanism followed by an abrupt change in fault geometry^36^ pointing to the existence of asperity.

The delineated asperity to the west of the epicentre requires special mention in view of the seismic hazard of the region. The area covered by this asperity is about 57% that of the eastern counterpart (Fig. 4, Supplementary Fig. S11). Assuming a 3 m average slip, as in the case of 2015 Gorkha event, the western asperity has the potential to generate an earthquake of magnitude M_w_ ~ 7.6. However, owing to complexities in rupture propagation to regions of residual strain, larger moments and more heterogeneous slip patterns can arise contrary to those expected based on typical scaling laws^7,30^. Hence, it is reasonable to envisage that a rupture due to the western asperity can have a magnitude close to that of 2015 Gorkha event. Coseismic stress induced by the 2015 Gorkha event and absence of afterslip towards west of the epicentre may further enhance the probability of rupturing the western asperity in future.

Another important aspect related to seismic potential and hazard of the Himalayas is to understand the role of incomplete ruptures. Based on geodetic and paleoseismic data, it is proposed that strain imparted by the partial ruptures may trigger larger events resulting in ruptures reaching the MFT^37^. The 2015 Gorkha earthquake occurred in a ‘seismic gap’ region, between the 1505 and 1934 earthquakes rupture areas^38^ which have an estimated magnitude M > 8. Among the other past earthquakes that seem to have occurred in this gap, the 1344 event is believed to have ruptured the MFT (Fig. 4). Considering a 20 mm/yr convergence rate for this region, the potential slip estimates reach values as high as 13.5 m. However, the 1833 earthquake along with the 2015 Gorkha earthquake and a probable future rupture associated with the western asperity can together account only a fraction of the accumulated elastic strain energy. Thus, these partial ruptures may catalyse a future great earthquake by unlocking the shallow locked portion of the MHT.

## Methods

### GPS data processing

Continuous observation of GPS data from permanent sites provide opportunity to access crustal deformation in detail and reduce the amount of time to distinguish the tectonic strain signals. We have used the GPS data from 36 permanent stations of Nepal Geodetic network installed by Caltech, Tectonic observatory which are available through UNAVCO (http://www.unavco.org) and processed theses continuous data over Nepal Himalaya spanning the time period from 1996 to 2015 with the GAMIT/GLOBK software^39^. Global Mapping Function model was used for the tropospheric mapping function and Hydrostatic delay^40^. First order ionospheric effects are removed by means of the ionosphere-free linear combination. Solid earth and Pole tidal effects were corrected following IERS 2010 conventions while ocean tide loading effects were corrected using ocean tide model FES2012. In the second step, the loosely constrained estimates of these parameters and their daily covariance were used as quasi-observations and combined with global analysis of IGS data performed by Scripps Orbit and Permanent Array Centre (SOPAC). The GPS time series of all stations were corrected for antenna and coseismic offsets followed by outlier detection and removal based on post-fit residual deviations from 3 times RMS^41^. The resulting time series is detrended and reduced from atmospheric and hydrological loading using National Center for Environmental Protection (NCEP) and Gravity Recovery and Climate Experiment (GRACE) models respectively. The velocity at each sites were estimated in International Terrestrial Reference Frame 2008 (ITRF2008). To understand the state of deformation in local scale, we derived the residual velocity in Indian Reference Frame (IRF) by removing the Indian plate Euler pole and angular velocity^42^. The GPS velocities derived from the permanent network was compiled with all available data from literature after converting to the common IRF (Supplementary Fig. S1-a, Supplementary Table S1). GPS time series showing daily position solutions and corrections applied for selected continues GPS stations are shown in Supplimentary Figs. S1-b and S1-c, respectively.

### InSAR data processing

ALOS-1 PALSAR data during 2007–2011 period acquired in the central Himalaya was utilized to map interseismic velocity field. The Himalayan collision zone is covered with three frames along the ascending tracks of the ALOS satellite (Supplementary Fig. S2-a). Interferograms are generated using GMTSAR software^43^ and unwrapped using SNAPHU algorithm^44^. The topographic phases were removed using Shuttle Radar Topographic Mission (SRTM) data. The residual phases were converted to Line of Sight (LOS) displacement and geocoded to produce the deformation maps. A total number of 100 interferograms were generated. After detailed analysis of interferometric coherence and phase unwrapping errors, 12–15 interferograms having baselines less than 450 m from each frame are selected (Supplementary Table S2) for time series analysis using Small Baseline Subset Interferometry (SBAS) techniques^45^ as implemented in GIANT software^46^. Atmospheric corrections to the InSAR data was were carried out using ECMWF weather model^47^. We have processed InSAR data separately for each frame along the satellite track. Subsequently, unwrapped phases are stitched together using a cosine weighted average function as implemented in GMT. Sample unwrapped interferograms and cumulative deformation maps with respect to the first observation date are presented as Supplementary Figs. S2-b and S2-c, respectively. The uncertainty correspondence to each pixel and epoch of the ALOS interferogram is determined using a “jackknife” approach. The uncertainty range of the ALOS InSAR velocity is estimated as 0.5–3.5 mm/yr. This is comparable with the uncertainty range obtained for the ENVISAT InSAR velocity data^14^ (0.7–3.1 mm/yr). We subsample the ALOS and ENISAT derived deformation to 44 and 112 points using a quad-tree method for the purpose of inversion.

### Joint inversion of geodetic data

In order to estimate the spatial variations in the slip rate and interseismic coupling, we invert geodetic dataset assuming linear superposition of rectangular dislocations in elastic half-space^18^. We use SDM (steepest descent method) iterative algorithm^48^ for the constrained least squares optimization to solve for the dip-slip and strike-slip components. In the inversion, the long-term slip rate is set as 20 mm/yr based on the geological evidences^12^, and allows us to compute slip deficit envisaged in the back slip model^49^. Finally, the interseismic slip rate is converted to ISC using the following formula

1 $$ISC=\frac{v^{\prime} -v}{v^{\prime} }$$

where, *v* is the interseismic slip rate and v′ is the long-term slip rate (20 mm/yr) imposed by plate convergence. In principle, in absence of transient slip events along the MHT, interseismic coupling should be between 0 and 1. ISC = 0 indicates that the patch creeps at the long term slip rate, whereas ISC = 1 indicates that the patch is locked.

#### Fault geometry and modelling procedure

The fault is divided into 742 rectangular patches of size of about 19 km^2^, for each of which the slip is computed. The dip of the fault is set to 10° towards north from the MFT. The curved shape of the MFT is approximated with 15 along strike fault segments with an average strike angle of 286°. The optimum smoothing factor (γ^2^ = 0.225) for the inversion was chosen from the trade-off curve between the model roughness and misfit (Supplementary Fig. S4-a). The optimum weighting ratio between GPS and InSAR-leveling data $(\alpha =\tfrac{Wight\,(GPS)}{Weight\,(InSAR\,\& \,Leveling)})$ is chosen as 4 based on the trade-off curve between α and solution misfit (Supplementary Fig. S4-b). Fit between observed and modelled GPS and InSAR/levelling are provided in Fig. 3 and Supplementary Fig. S5, respectively.

#### Resolution tests

We have carried out resolution tests to assess the efficacy of ISC retrieval by inverting synthetic geodetic data generated from reference ISC distribution representing asperities along the fault plane. We have tested two simple asperity models consisting of locked fault patches (ISC = 1) of widths 70 and 100 km, embedded in free slipping surface (ISC = 0). In both the models, the asperity is defined as locked from surface to 20 km depth. GPS, InSAR and levelling velocities are simulated at observation points using forward computations. We add random noise to the modelled velocities and reinvert for the optimal slip distribution (Supplementary Fig. S6). The random noise are computed from the mean and standard deviations of the measurement uncertainties and follows Gaussian distribution. Smoothing factor for the inversion was chosen from the trade-off curve between the model roughness and misfit. The sensitivity of the inversion to the addition of noise is tested by systematically increasing the amplitude of added noise.

### Total strain rate estimation

To visualize the state of deformation, we estimated the horizontal strain rate pattern from the GPS derived velocity vectors by a weighted least square method^50^. The contribution of each station velocity to the strain rate computed on a given node of the grid is weighted by using the optimal interpolation method^50,51^. In order to relate the strain rate to the interseismic seismicity, independently from the principal strain components, we have estimated the total strain rate (i.e. 2^nd^ invariant of strain) from the horizontal components of the strain rate tensor^52^. Quality of the strain rate is expressed as Signal to Noise Ratio map generated by dividing the second invariant with its standard deviation (Supplementary Fig. S8). The SNR map largely reflects the spatial density of the geodetic stations and thus low SNR regions typically correspond to regions having large gaps in GPS stations. The central region (84° to 87°E) has high SNR providing confidence to the interpretation of the high strain rate patches identified in the present study (Supplementary Fig. S8).

### b-value estimation

We use microseismicity data^29^ of the Nepal region acquired during 1994–2009 by NSC for the b-value estimation. In order to suppress the contamination from aftershocks, the catalogs were declustered^29,53^, using temporal separation 1 ≤ *τ* ≤ 10 days, inter-event separation (*D*) = 20 km, horizontal separation (*U*_*x*_) = 5 km, vertical separation (*U*_*z*_) = 10 km and confidence level *P* = 0.95. From about 22000 events, we retain events shallower than 35 km and spatially falling within the boundary of the MHT between 82°–88°E longitude for the analysis (Supplementary Fig. S9). By doing so, we eliminate deep crustal events that are not associated with mid-crustal seismicity and plate-coupling processes related to the MHT. We perform the frequency-magnitude analysis of the data using maximum curvature method provided in the software ZMAP^54^. The magnitude of completeness (*M*_*c*_) was determined as 2.4 using the iterative method^55^. We compute *b-value* from ~7200 events having magnitude > Mc using maximum likelihood method^56^. The estimation was done at each 0.05° grid points for closest 125 events in a vertical cylinder with a sampling radius of 25 km. We also compute the error in *b-value*, *Mc* at each grid node as 1-sigma standard deviation using 10,000 bootstrap simulations (Supplementary Fig. S10). For most of the regions, the computed errors are less than 10% of the associated b-values. Further, b-values were estimated for selected nodes independently using a least square method. Close match between these estimations with the b-value map further suggest the reliability of the spatial variations in the b-values (Supplementary Fig. S5-c). Further, we observe that *b-value* estimation at a larger grid size (0.1°), keeping all other parameters same, did not show any significant change in the pattern of observed b-values (Fig. S5-d).

### Code availability

Code used for 2D GPS profiles modelling is available with K.M.S. and S.P.S. Reference to other publically available software/codes are cited in the method section.

## Electronic supplementary material

Supplementary Information

## Data Availability

ALOS-1 InSAR data are available with the first author. Raw GPS data from Nepal Geodetic Network are freely downloadable from UNAVCO (http://www.unavco.org). Other published data sets are cited in main text/supplementary information.
