# Supplementary material for: Audit of stored strain energy and extent of future earthquake rupture in central Himalaya
Source: Sci Rep. 2018 Nov 12;8:16697. doi: 10.1038/s41598-018-35025-y (PMC6232156; doi:10.1038/s41598-018-35025-y)
Supplement: Supplementary file 1 — Supplementary Information [file 41598_2018_35025_MOESM1_ESM.docx]

**Supplementary Information**

**Audit of stored strain energy and extent of future earthquake rupture in central Himalaya**

K. M. Sreejith^*^, P.S. Sunil, Ritesh Agrawal, Ajish P. Saji, A.S. Rajawat and D.S. Ramesh

^1^Space Application Centre (ISRO), Ahmadabad, India

^2^Indian Institute of Geomagnetism (DST), Mumbai, India

^3^Department of Marine Geology and Geophysics,

Cochin University of Science and Technology (CUSAT), Kochi, India

[^*^sreejith81@gmail.com](mailto:*sreejith81@gmail.com)

**Electronic supplement:** 11 figures (S1-S11)

3 Tables (S1-S3)

***Table S1*** *Details of GPS data used for the analysis*

| No | GPS data source | Period | GPS operation |
| --- | --- | --- | --- |
| 1  2  3  4  5  6  7  8  9 | Nepal network  (present study)  Bettinelli et al., 2006  Liang et al., 2013  Ponraj et al., 2011  Mukul et al., 2010  Banerjee et al., 2008  Dumka et al., 2014  MulliK et al., 2009  Ader et al., 2012 | 1996-2015  1995-2004  1998-2013  1999-2007  2000-2004  2003-2006  2003-2006  2005-2008  2009-2012 | Continuous  Continuous & Campaign  Continuous & Campaign  Campaign  Continuous & Campaign  Campaign  Campaign  Campaign  Continuous |


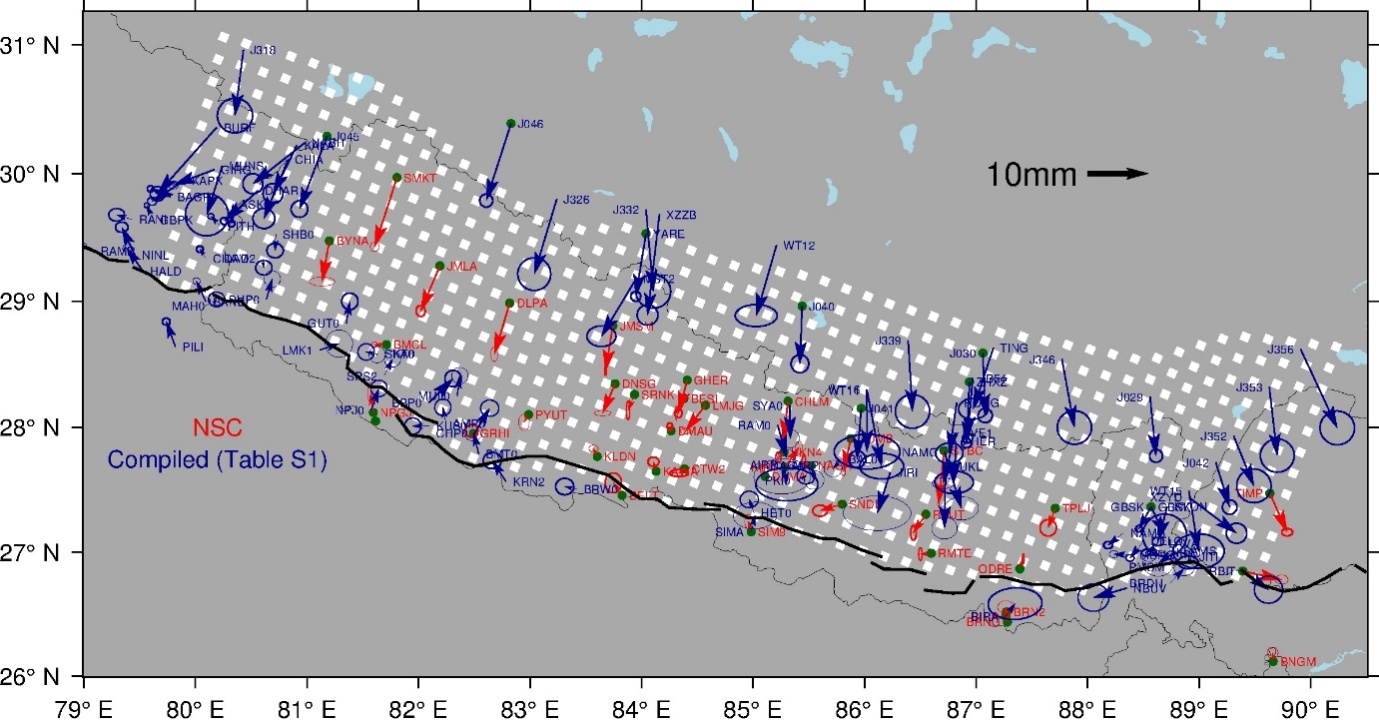


***Figure S1-a.*** *Map showing* *horizontal GPS velocity derived in the present study (red vector) and those compiled from literature (blue vectors) of central Himalaya. Green dots are locations of continues GPS data. Map view of the grid nodes of the model fault is shown as white boxes.*

***
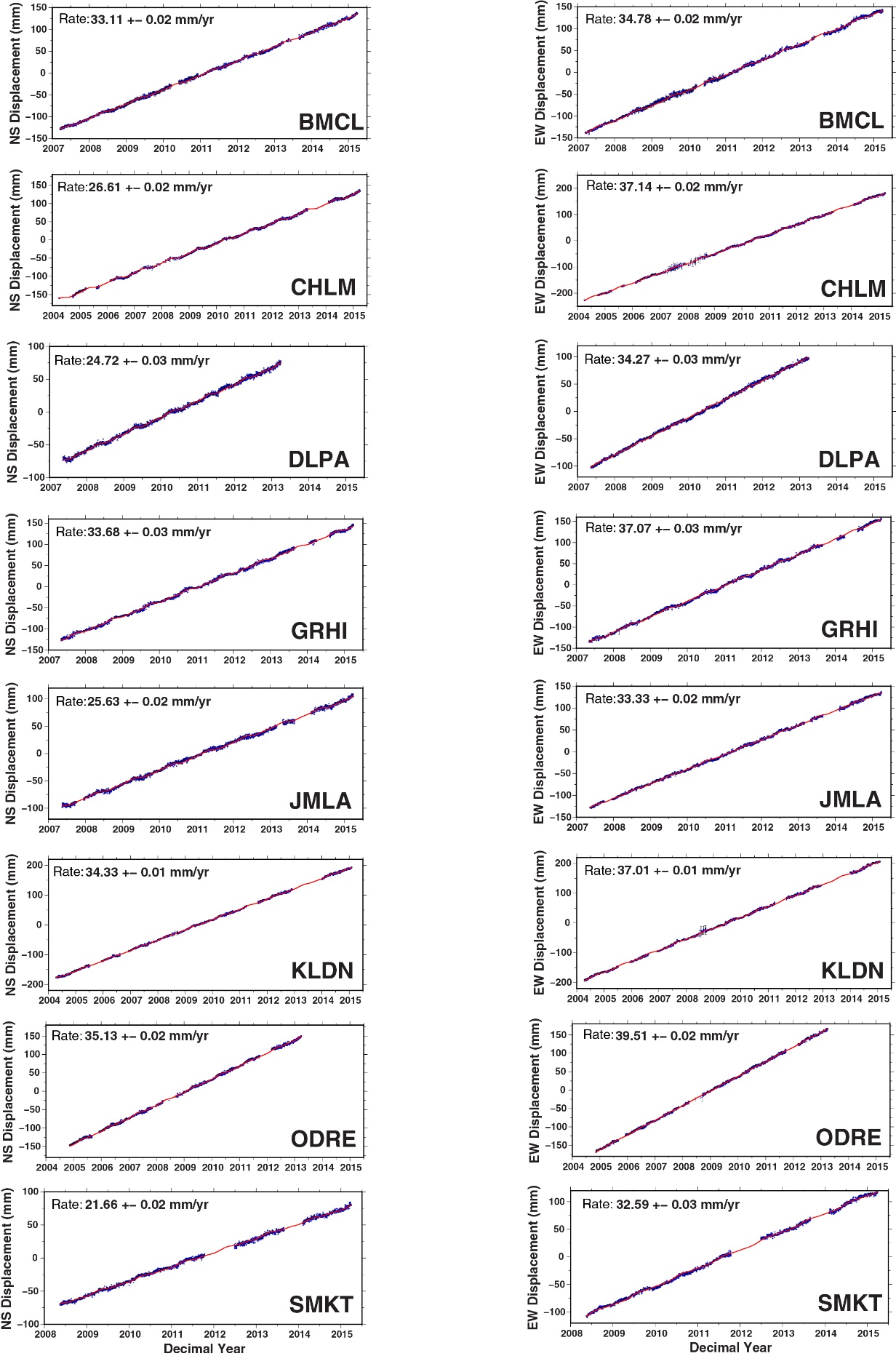
***

***Figure S1-b.*** *NS and EW components of daily solutions of positions as a function of time determined relative to ITFR2008 at 8 selected GPS sites from Nepal Geodetic network (blue dots). Red continues line shows the best-fit model for the estimation of velocity rate at each site. Time series displacements of these GPS sites are presented in Figure S1-c.*

***
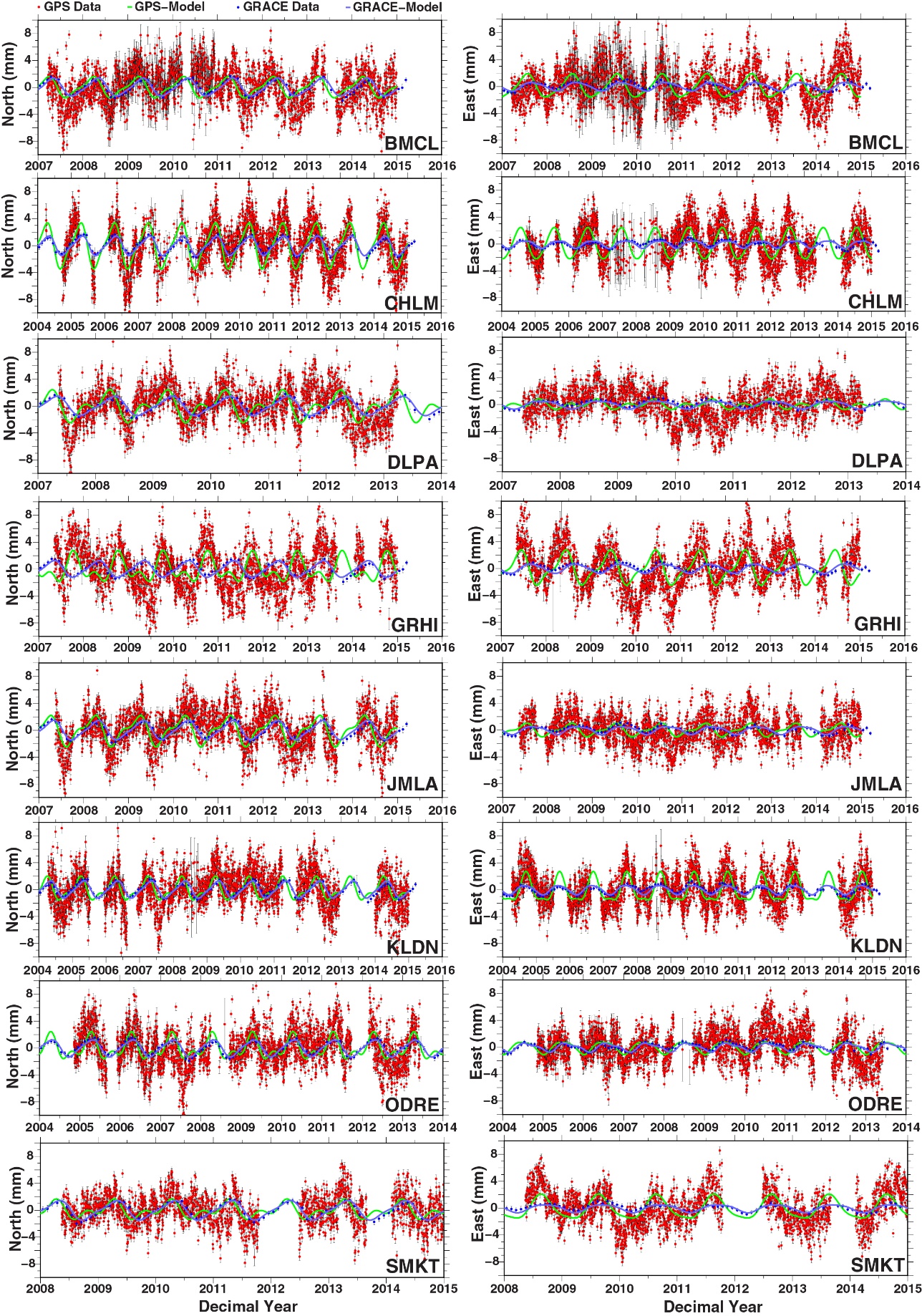
***

***Figure S1-c.*** *Time series plots showing detrended displacements of NS and EW components of the GPS sites (red dots). GRACE derived displacements at GPS sites are shown as blue circles. Modelled seasonal effects in GPS and GRACE time series are represented with green and blue lines, respectively.*


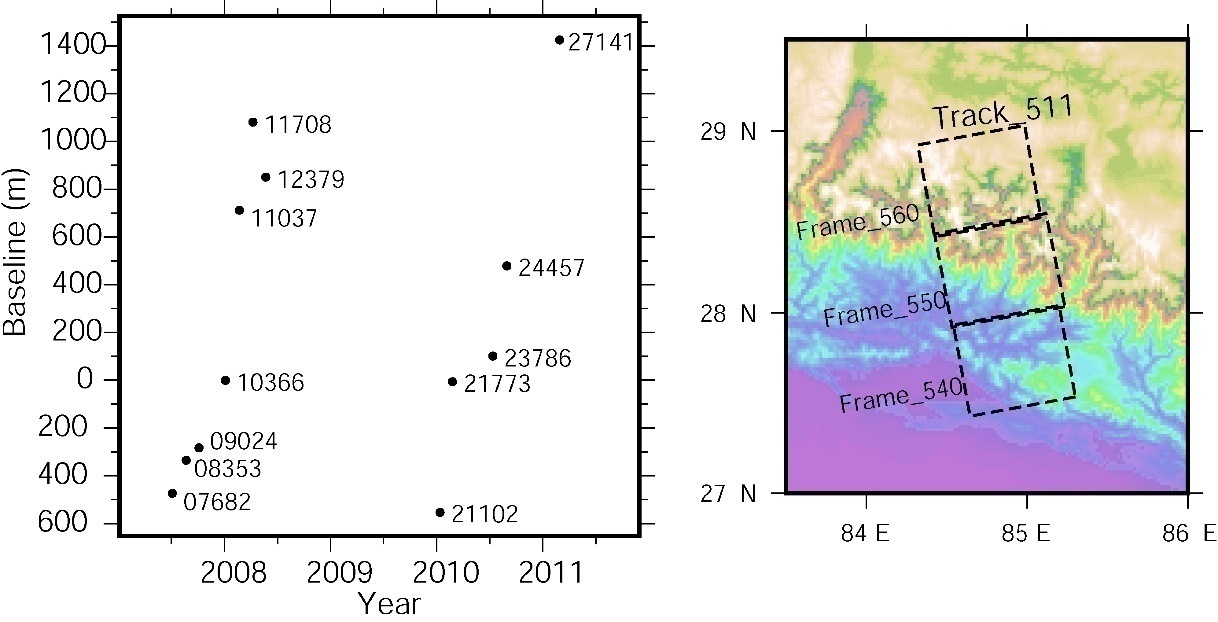


***Figure S2-a*** *Perpendicular baseline-time plot of ALOS data along track 511. The numbers written against ALOS data points are Scene ID. The position of the track and frames (540, 550 560) are shown in the right panel. Details of interferogram generated for each frame is provided in Table S2.*

*
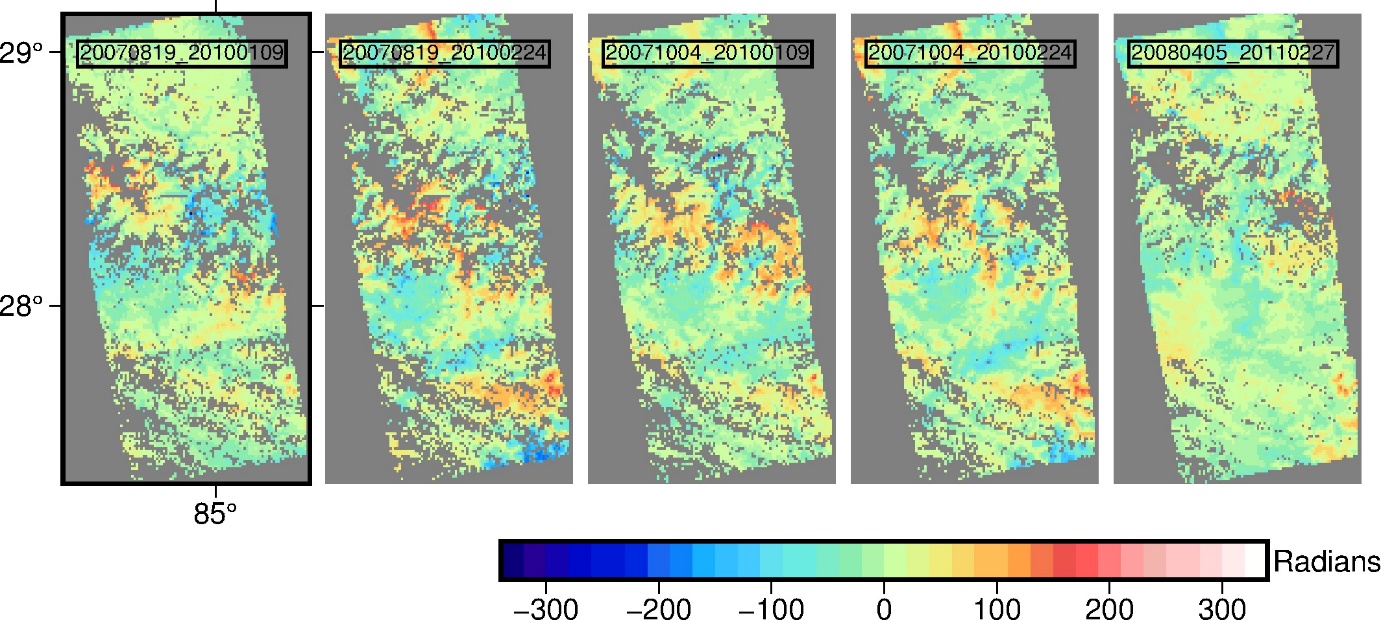
*

***Figure S2-b*** *Sample unwrapped interferograms. Interferometric pairs are named according to the date of data acquisitions.*

*
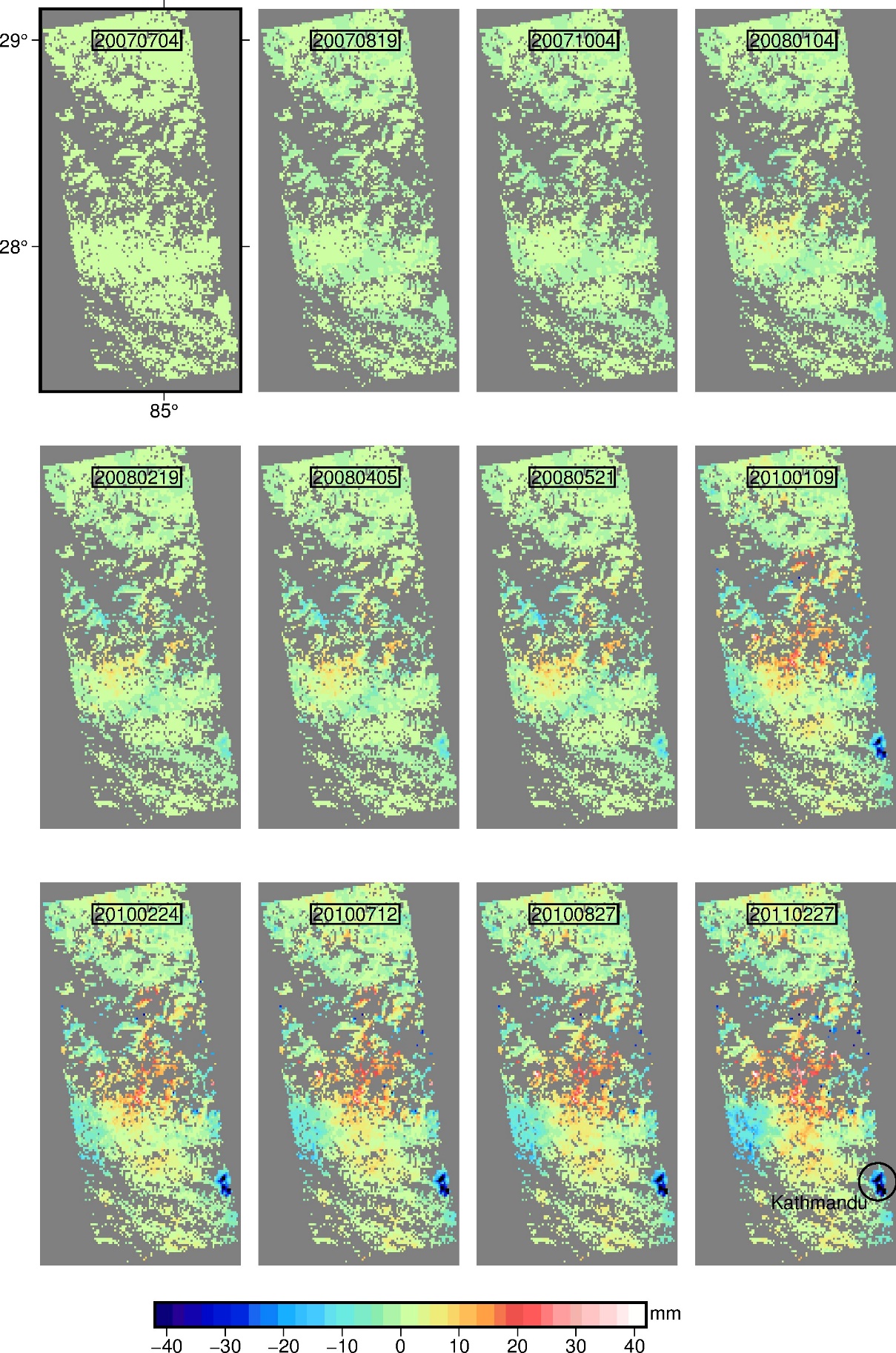
*

***Figure S2-c*** *Cumulative deformation maps with respect to the first date of observation. Large subsidence shown in the back circle is probably associated with non-tectonic deformation of Kathmandu basin related to groundwater withdrawal and those data points are avoided in the analysis.*

***Table S2*** *Details of ALOS interferograms and perpendicular baseline (B) used for the analysis.*

| **Frame 540** | | | **Frame 550** | | **Frame 560** | |
| --- | --- | --- | --- | --- | --- | --- |
| **No** | **Interferogram** | **B_per_ (m)** | **Interferogram** | **B_per_ (m)** | **Interferogram** | **B_per_ (m)** |
| 1  2  3  4  5  6  7  8  9  10  11  12  13  14  15 | 20070704_20100109  20070819_20100109  20070819_20100224  20071004_20100109  20071004_20100224  20071004_20100712  20080104_20100224  20080104_20100712  20080219_20100827  20080405_20110227  20080521_20100827  20100224_20100712 | \| -79.81 \| \| --- \| \| -217.54 \| \| 320.062 \| \| -258.64 \| \| 278.96 \| \| 371.19 \| \| 8.67 \| \| 100.90 \| \| -230.15 \| \| 322.21 \| \| -349.90 \| \| 92.23 \| | 20070704_20100109  20070819_20100109  20070819_20100224  20071004_20100109  20071004_20100224  20071004_20100712  20080104_20100224  20080104_20100712  20080219_20100827  20080405_20110227  20080521_20100827  20100224_20100712 | \| -78.46 \| \| --- \| \| -217.11 \| \| 325.09 \| \| -263.42 \| \| 278.78 \| \| 378.39 \| \| 1.80 \| \| 101.41 \| \| -232.11 \| \| 333.72 \| \| -359.81 \| \| 99.61 \| | 20070704_20100109  20070819_20100109  20070819_20100224  20071004_20100109  20071004_20100224  20071004_20100712  20080104_20070819  20080104_20071004  20080104_20100224  20080104_20100712  20080219_20080405  20080219_20080521  20080219 _20100827  20080405_20110227  20080521_20100827 | \| -77.10 \| \| --- \| \| -216.6 \| \| 330.09 \| \| -268.17 \| \| 278.59 \| \| 385.57 \| \| -335.17 \| \| -283.66 \| \| -5.07 \| \| 101.92 \| \| 365.92 \| \| 135.63 \| \| -234.06 \| \| 345.19 \| \| -369.69 \| |


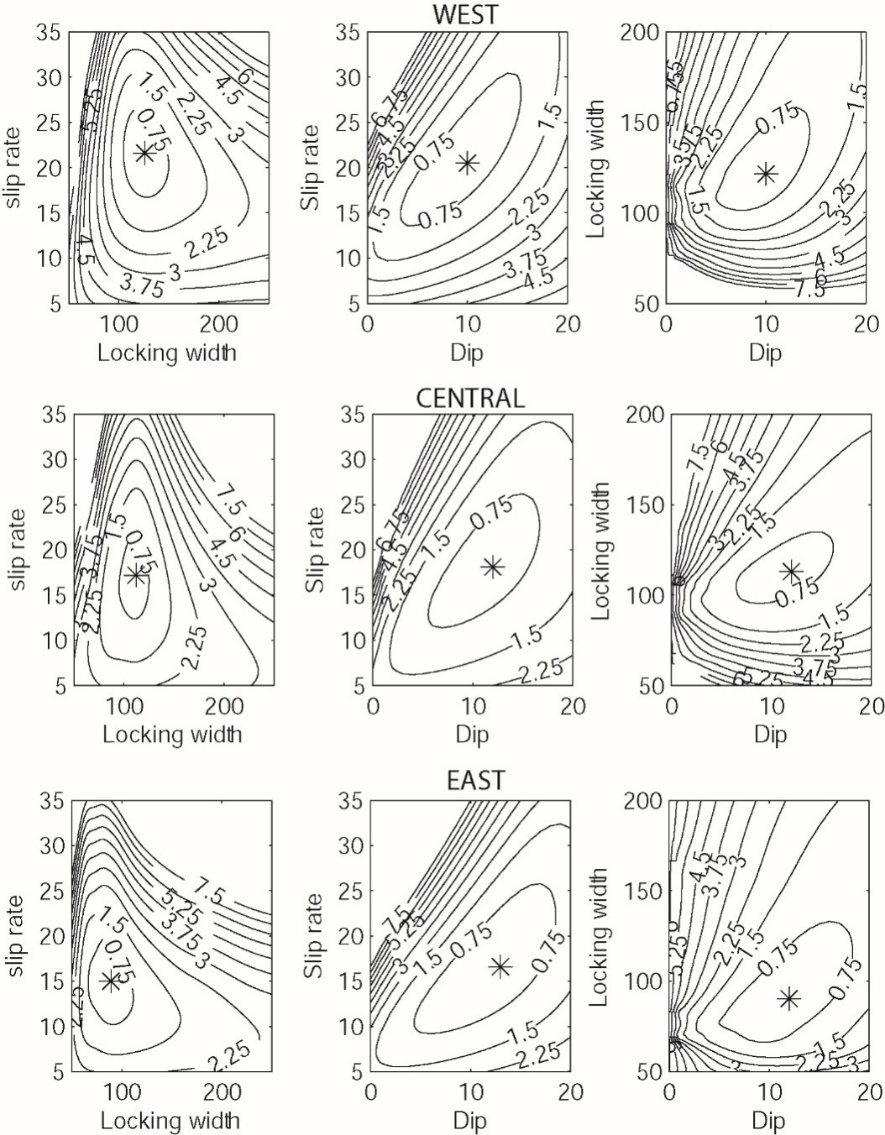


***Figure S3.*** *Chi-squared misfit (mm/yr) between observed and modelled GPS displacements contoured in the model parameter space for West, Centre and East GPS profiles.*

***Table S3.*** *Best-fit model parameters (Slip rate (S), Locking with (W) and Dip (d)) derived from the forward modelling of GPS profiles. Error bounds are calculated at 90% confidence interval.*

| **West** | | | **Central** | | | **East** | | |
| --- | --- | --- | --- | --- | --- | --- | --- | --- |
| Slip rate  (mm/yr) | Locking width  (km) | Dip  (degree) | Slip rate  (mm/yr) | Locking width  (km) | Dip  (degree) | Slip rate  (mm/yr) | Locking width  (km) | Dip  (degree) |
| 21.6±1.7 | 126±7.6 | 10±2.2 | 17.2±2.1 | 112±5.8 | 12±2.4 | 15.1±1.4 | 90±5.5 | 12±2.1 |


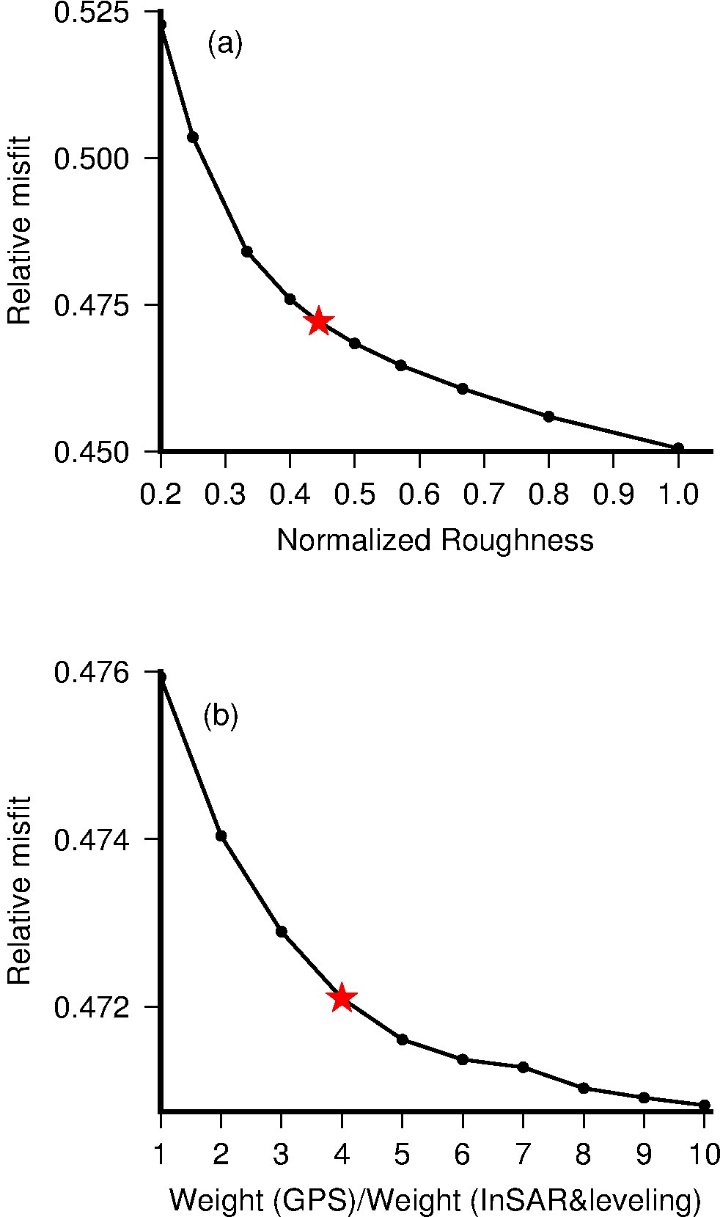


***Figure S4 a,*** *Trade-off curve (L-curve) showing the ratio of the model misfit against the model roughness. The bending point of the curve (red star) corresponds to the preferred roughness value.* ***b****, Model misfit-relative weight curve. The preferred weight used for the inversion is indicated with red star.*

a)


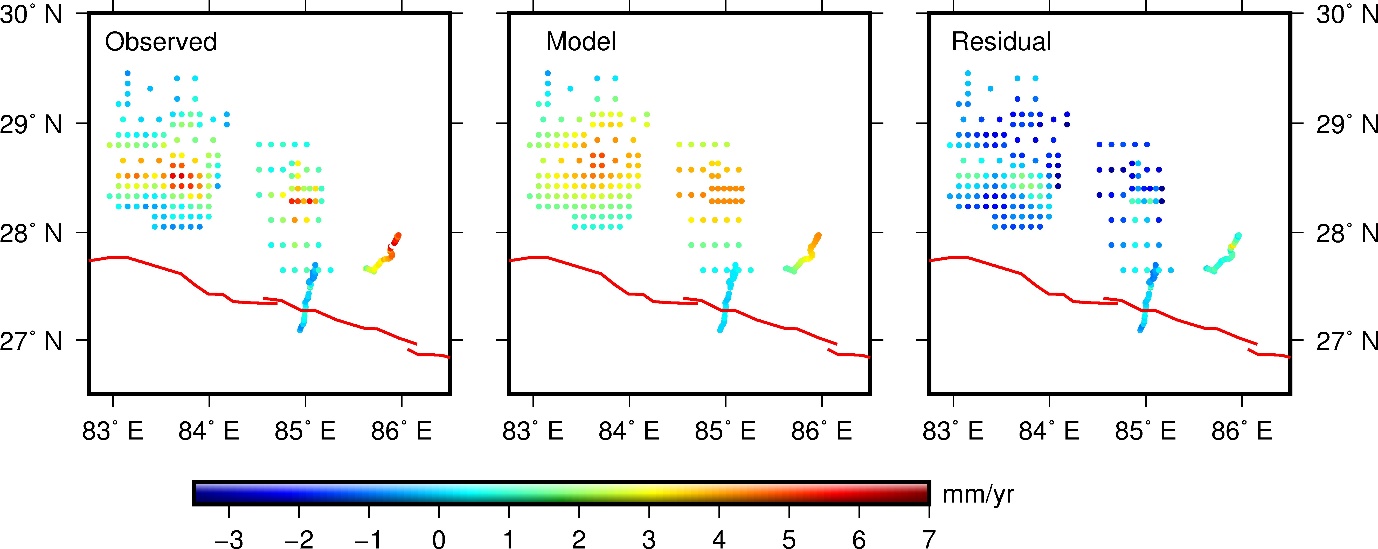


b)


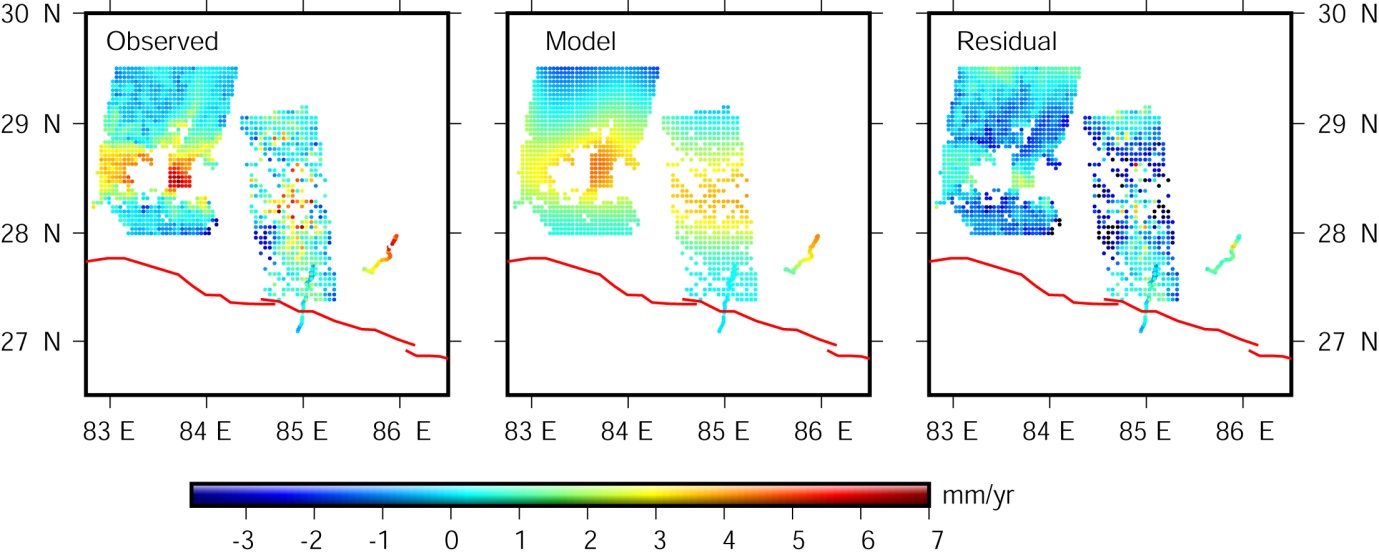


**Figure S5 a)** *Model fit for the quad-tree sampled InSAR and Levelling data. b) Model fit for the uniformly sampled InSAR data.*

**
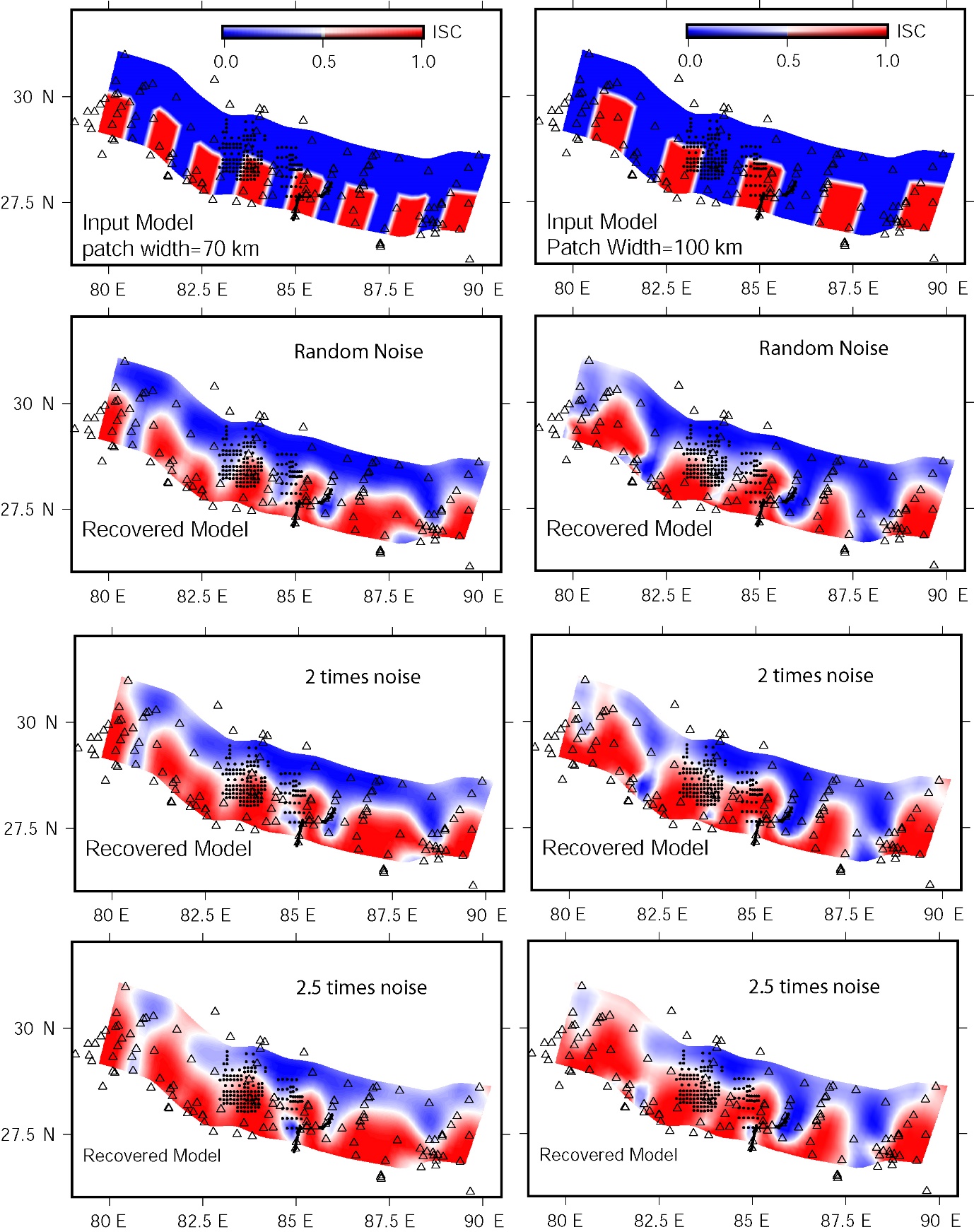
**

***Figure S6.*** *Resolution test of the inversion for ISC. (Left) ISC patches (~70 km) are effectively resolved between 82°E – 85.5°E, where InSAR and levelling data are available. (Right)* *Input patches with width ~ 100 km are resolved throughout the fault plane. The lower two panels shows the effect of random Gaussian noise added before inversion. When the noise level is increased to 2.5 times input patches are not recovered.*

*
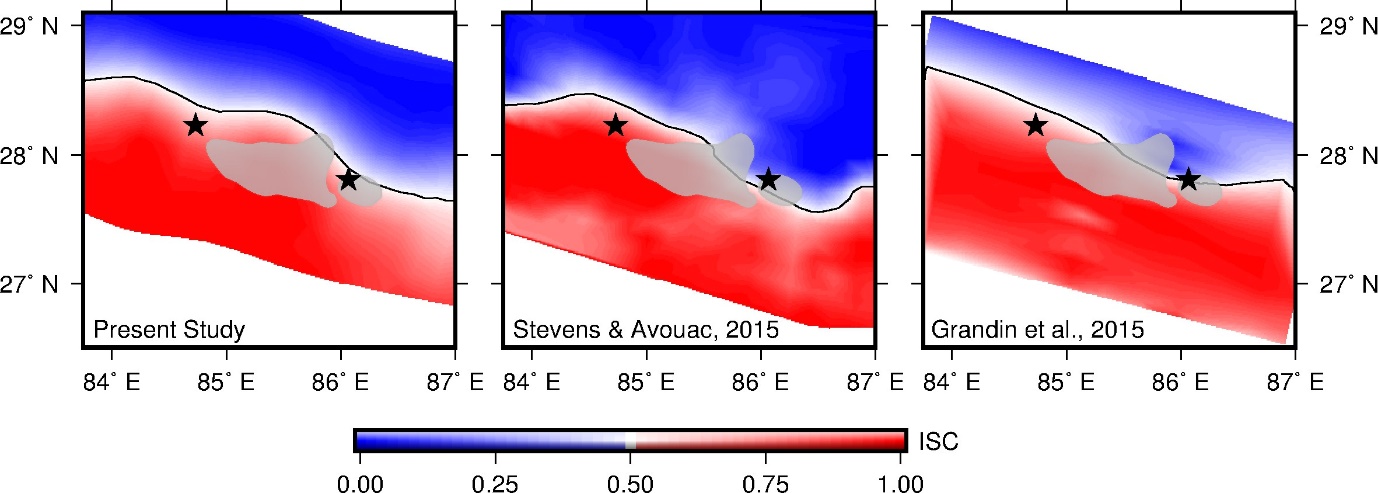
*

***Figure S7*** *Comparison of ISC pattern obtained in the present study with that of Stevens and Avouac (2015) and Grandin et al, (2015). Epicentre locations of the 2015 Gorkha earthquake and aftershock M_w_=7.3 are shown as black stars. Rupture area of the earthquakes are shaded. ISC=0.5 contour is also shown as black line.*

**
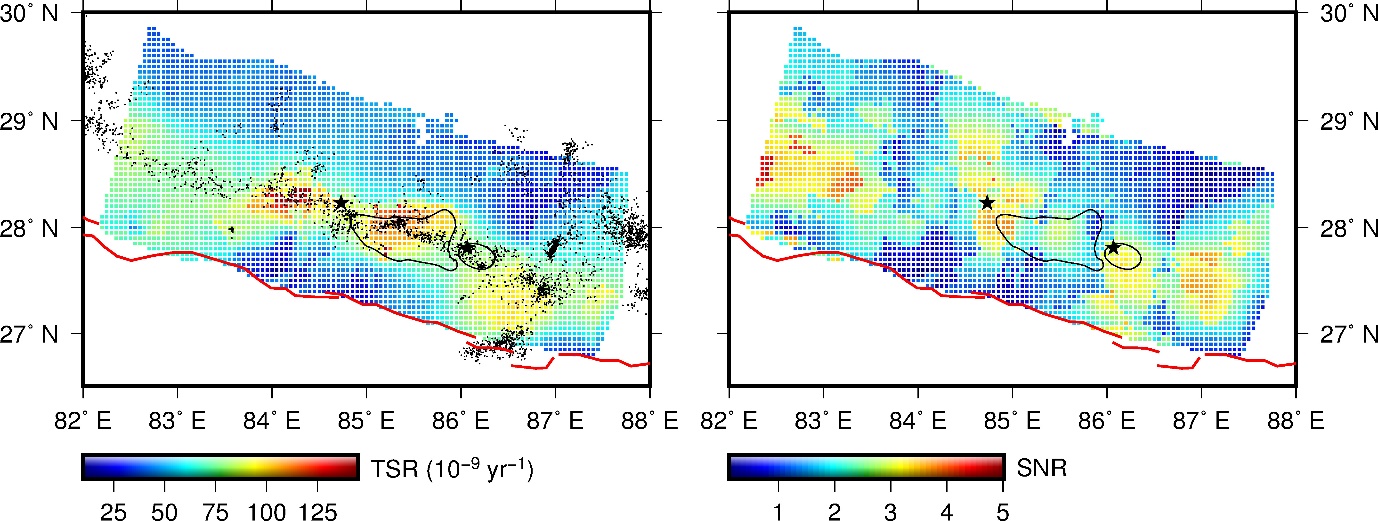
**

***Figure S8*** *Maps of total strain rate and signal to noise ratio. Epicentre locations of the 2015 Gorkha earthquake and aftershock M_w_=7.3 are shown as black stars. Rupture area of the earthquakes are shown as black line. Locations of microseismicity data is plotted as black dots.*

**
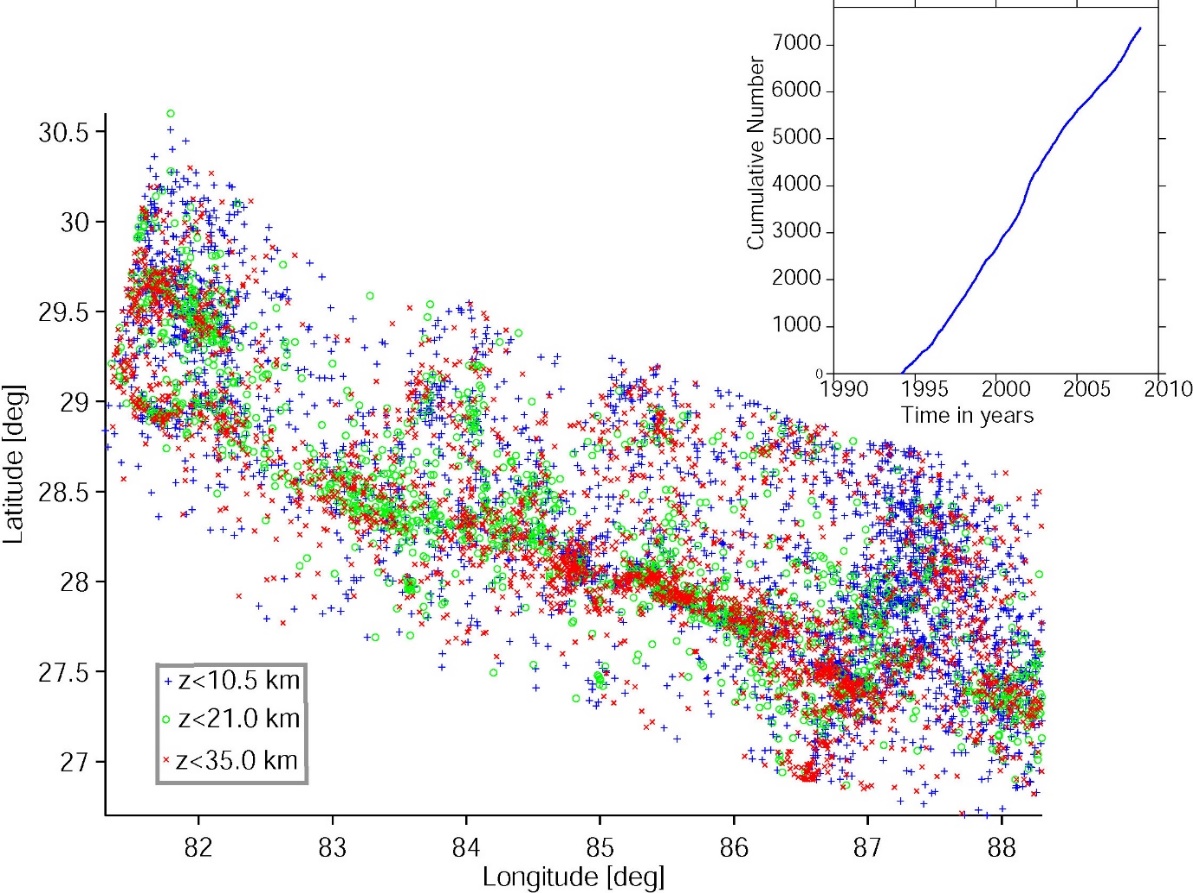
**

***Figure S9*** *Seismicity data (M>Mc) used for the b-value estimation. The insert plot shows the cumulative earthquake distribution over time.*

d)

**
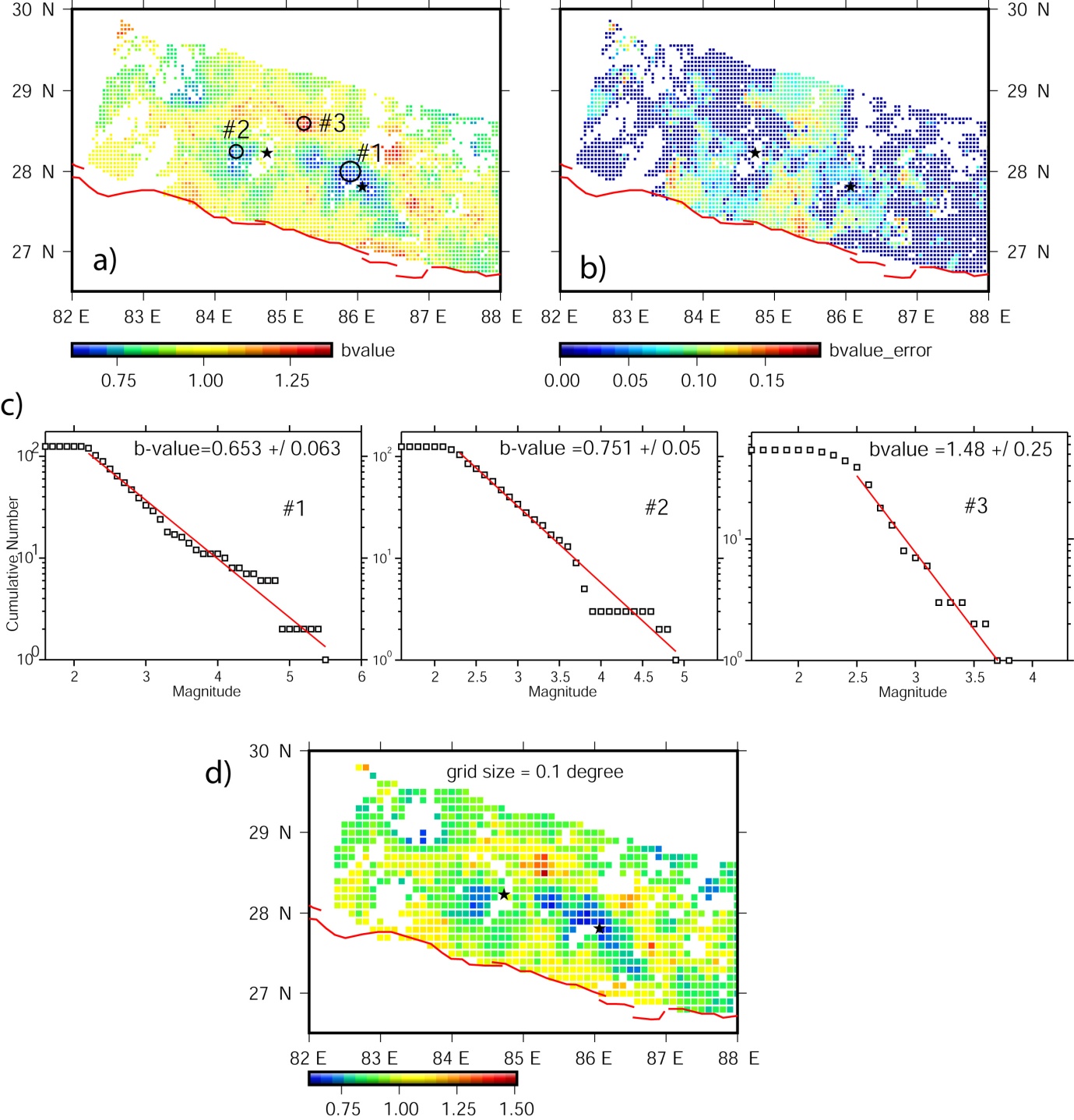
**

***Figure S10 a-b,*** *Maps of b-value and error in b-value estimates.* ***c,*** *frequency magnitude distribution and b-value estimates corresponding to regions #1, #2 and #3 indicated by black circles. d) b-value estimation using larger (0.1-degree) grid size. Epicentre locations of the 2015 Gorkha earthquake and aftershock Mw=7.3 are shown as black stars.*

**
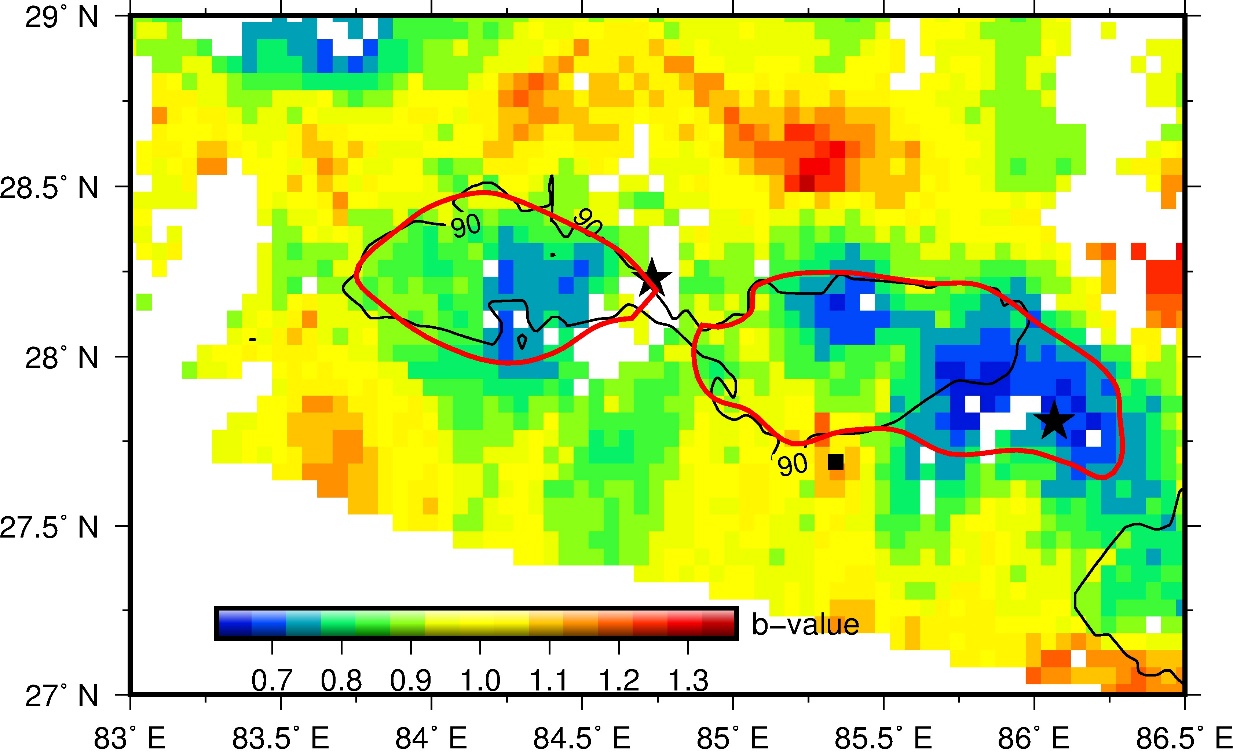
**

**Figure S11** *Subset of b-value map and total strain rate contour corresponding to 90×10^-9^yr^-1^ (Black line). The extent of asperities (Figure 4) are outlined by red line. Epicentre locations of the 2015 Gorkha earthquake and aftershock Mw=7.3 are shown as black stars.*

**References**

1. Bettinelli, P., Avouac, J. P., Flouzat, M., Bollinger, L., Ramillien, G., Rajaure, S., & Sapkota, S. (2008). Seasonal variations of seismicity and geodetic strain in the Himalaya induced by surface hydrology. *Earth and Planet. Sci. Lett*., **266(3)**, 332-344.
2. Liang, S. et al. Three-dimensional velocity field of present-day crustal motion of the Tibetan Plateau derived from GPS measurements, *J. Geophys. Res*. **118**, 5722-5732 (2013).
3. Ponraj, M., Miura, S., Reddy, C.D., Amirtharaj, S. & Mahajan, S.H., Slip distribution beneath the Central and Western Himalaya inferred from GPS observations, *Geophys. J. Int.* **185**, 724-736 (2011).
4. Mukul, M. First-order kinematics of wedge-scale active Himalayan deformation: Insights from Darjiling–Sikkim–Tibet (DaSiT) wedge. *J. Asian. Earth. Sci.* **39**, 645-657 (2010).
5. Banerjee, Burgmann, R. Nagarajan, B. & Apel, E. Intraplate deformation of the Indian subcontinent, Geophys. Res. Lett. **35**, L18301 (2008).
6. Dumka R. K., Kotlia, B. S., Kumar, K. & Satyal., G. S. Quantification of crustal strain rate in Kumaun Himalaya (India) using GPS measurements of crustal deformation, *Himalayan Geology* **35**, 146-155 (2014).
7. Mullick, M., Riguzzi, F., & Mukhopadhyay, D. Estimates of motion and strain rates across active faults in the frontal part of eastern Himalayas in North Bengal from GPS measurements. *Terra Nova* **1**, 410-415 (2009).
8. Ader, T. et al. Convergence rate across the Nepal Himalaya and interseismic coupling on the Main Himalayan Thrust: implications for seismic hazard. *J. Geophys. Res*. **117,** B044403 (2012).
